# A Multi-Criteria Assessment Strategy for 3D Printed Porous Polyetheretherketone (PEEK) Patient-Specific Implants for Orbital Wall Reconstruction

**DOI:** 10.3390/jcm10163563

**Published:** 2021-08-13

**Authors:** Neha Sharma, Dennis Welker, Soheila Aghlmandi, Michaela Maintz, Hans-Florian Zeilhofer, Philipp Honigmann, Thomas Seifert, Florian M. Thieringer

**Affiliations:** 1 Clinic of Oral and Cranio-Maxillofacial Surgery, University Hospital Basel, CH-4031 Basel, Switzerland; neha.sharma@usb.ch (N.S.); hans-florian.zeilhofer@usb.ch (H.-F.Z.); 2 Medical Additive Manufacturing Research Group (Swiss MAM), Department of Biomedical Engineering, University of Basel, CH-4123 Allschwil, Switzerland; dennis.welker@gmail.com (D.W.); michaela.maintz@unibas.ch (M.M.); philipp.honigmann@ksbl.ch (P.H.); 3 Basel Institute for Clinical Epidemiology and Biostatistics, Department of Clinical Research, University Hospital Basel, CH-4031 Basel, Switzerland; soheila.aghlmandi@usb.ch; 4 Institute for Medical Engineering and Medical Informatics, University of Applied Sciences and Arts Northwestern Switzerland, CH-4132 Muttenz, Switzerland; 5 Hand Surgery, Cantonal Hospital Baselland, CH-4410 Liestal, Switzerland; 6 Department of Biomedical Engineering and Physics, Amsterdam UMC, University of Amsterdam, Amsterdam Movement Sciences, NL-1105 Amsterdam, The Netherlands; 7 Department of Mechanical and Process Engineering, University of Applied Sciences, DE-77652 Offenburg, Germany; thomas.seifert@hs-offenburg.de

**Keywords:** blow-out, biocompatible materials, computer-aided design, finite element analysis, orbit, implant, orbital fracture, patient-specific modeling, printing, three-dimensional

## Abstract

Pure orbital blowout fractures occur within the confines of the internal orbital wall. Restoration of orbital form and volume is paramount to prevent functional and esthetic impairment. The anatomical peculiarity of the orbit has encouraged surgeons to develop implants with customized features to restore its architecture. This has resulted in worldwide clinical demand for patient-specific implants (PSIs) designed to fit precisely in the patient’s unique anatomy. Material extrusion or Fused filament fabrication (FFF) three-dimensional (3D) printing technology has enabled the fabrication of implant-grade polymers such as Polyetheretherketone (PEEK), paving the way for a more sophisticated generation of biomaterials. This study evaluates the FFF 3D printed PEEK orbital mesh customized implants with a metric considering the relevant design, biomechanical, and morphological parameters. The performance of the implants is studied as a function of varying thicknesses and porous design constructs through a finite element (FE) based computational model and a decision matrix based statistical approach. The maximum stress values achieved in our results predict the high durability of the implants, and the maximum deformation values were under one-tenth of a millimeter (mm) domain in all the implant profile configurations. The circular patterned implant (0.9 mm) had the best performance score. The study demonstrates that compounding multi-design computational analysis with 3D printing can be beneficial for the optimal restoration of the orbital floor.

## 1. Introduction

Pure orbital blowout fractures, also known as internal orbital floor fractures, occur within the confines of the internal orbital wall and do not affect the orbital rim or other facial bones. These fractures are common in individuals who experience blunt trauma to the facial and skull region [1,2,3]. The treatment of orbital blowout fractures is challenging mainly due to the restricted intraoperative view of the intricate and delicate anatomical region. Restoration of orbital form and volume is paramount to prevent functional and esthetic impairment [4,5].

Currently, the conventional methods of orbital floor fracture reconstruction include the use of permanent, alloplastic non-resorbable biomaterials such as titanium meshes (prebend or prefabricated), plates, or polymeric implants (polyethylene, with embedded titanium mesh) [6,7]. Porous polyethylene implants are malleable and allow vascular ingrowth due to open pore structure. However, if placed close to extraocular muscles, it may form adhesions [8,9]. When utilizing conventional titanium meshes, pre- or intraoperative bending and shape adjustment are required, making the correct placement and location of the implants within the orbit a challenge. A typical issue associated with improperly positioned pre-bent plates is a lack of distal or medial support caused by damage to the orbital ledge and/or intra-orbital buttress [10,11,12]. The placement of orbital implants and their size and shape conformance to the unique anatomy of the injured components are critical factors in the overall success rate of orbital reconstruction, which has prompted surgeons to develop novel treatment options for orbital floor reconstructions [13,14].

With advances in computer-assisted surgery (CAS), orbital reconstructions have witnessed tremendous progress, dramatically improving both the functional and esthetic outcome of reconstructions with customized implants [15,16]. The customized or patient-specific implants (PSIs) enable patient-centric surgical plans and achieve precise orbital reconstructions [17,18,19,20]. Along with meticulous virtual surgical planning (VSP) and clinical examination, another pivotal component in treating orbital floor fractures is selecting the reconstructive material [21,22,23].

Over the last years, Polyetheretherketone (PEEK), a high-performance polymer, has gained significant popularity in reconstructive surgeries [24,25,26,27]. PEEK possesses a bone-like modulus of elasticity, excellent biocompatibility, high yield strength, and fatigue resistance, making it an appealing biomaterial for personalized implants in craniomaxillofacial surgery [28,29,30]. The adoption of PEEK for PSIs production was influenced by its favorable properties, including radiolucent characteristics with no artifact in medical imaging, stiffness, lightweight, and conventional computer-aided design/computer-aided manufacturing (CAD/CAM) procedures, specifically milling [25,30]. Few studies have reported using custom-made, non-porous, milled PEEK implants in orbital reconstructions [19,31,32]. However, these non-porous characteristics displaying hydrophobicity and bio-inertness of PEEK can limit its bioactivity and cause clinical concern in orbital floor reconstructions [33,34].

Numerous attempts have been carried out to enhance the osseointegration potential of PEEK with surface coatings. Nonetheless, degradation and inadequate binding of the coatings to the PEEK implant surface resulting in osteolysis have been reported [35,36,37]. Another strategy is the introduction of porous structures, which has demonstrated promising results in increasing the osteogenic potential of PEEK [38,39,40]. The methods of fabricating three-dimensional (3D) porous structures are limited in conventional manufacturing technologies, and therefore, the clinical interest in additive manufacturing (AM) or 3D printing has rapidly grown [35,38,41,42].

The anatomical peculiarity of the orbital region has encouraged surgeons to develop implants with customized characteristics to restore orbit architecture. This has resulted in worldwide clinical demand for PSIs designed to fit precisely in the patient’s unique anatomy. With improvement in AM systems, the potential for customized 3D printed PEEK customized implants has surfaced, boosting interest in point-of-care (POC) manufacturing. Material extrusion-based or fused filament fabrication (FFF) 3D printing technology has previously been confined to low-temperature thermoplastics; however, the latest advances have enabled printing of high-temperature, implantable-grade thermoplastic polymers such as PEEK, paving the way for a more sophisticated generation of biomaterials. Implementing FFF at the POC offers numerous advantages such as less material wastage, easy operator training, faster implant production, increased cost-effectiveness, and patient specificity [25,26,29,43].

In orbital floor reconstruction, the implant’s perfusion, permeability, and adequate mechanical strength are critical for long-term clinical success [21,44]. The characteristics of a porous implant are crucially dependent on its structure, which can significantly impact the mechanical response, defining its clinical applicability. Additionally, the design freedom capabilities of computer-aided design modeling and 3D printing significantly increase the possible combinations for an implant, resulting in various treatment choices for a specific case [42]. The design of porous constructs and implant thicknesses can thus affect the performance of orbital mesh implants. An essential factor to consider in such scenarios is the load-bearing capability of an implant under physiological conditions. From the biomechanical point of view, analyzing the implant’s stress and deformation patterns can help understand better how the PEEK orbital mesh implants might respond to the orbital floor reconstruction regime. In this regard, computational models and simulations provide an estimate of the load-bearing capacity of the design before the fabrication of an implant [45,46]. Lastly, defects arising from the printing process and inadequate post-processing processes can significantly impact an implant’s structural integrity and robustness. The printing feasibility and morphological characteristics of the respective implant dictate its conclusive clinical appropriateness and applicability.

There are currently no studies that provide an insight into the POC FFF 3D printing of PEEK orbital mesh customized implants. Therefore, this study aims to evaluate the performance of the FFF 3D printed PEEK orbital mesh implants with a metric considering the relevant design, biomechanical, and morphological parameters. To provide deeper insights, the complete in-house digital workflow from the pre-operative VSP to mechanical characteristics and FFF production of PEEK orbital mesh customized implants is studied as a function of varying thicknesses and porous design constructs through a finite element (FE) based computational model and a decision matrix based statistical approach.

## 2. Materials and Methods

**Figure 1:**
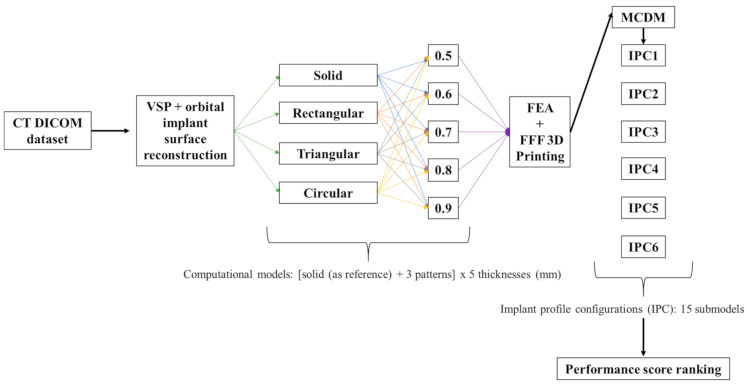
Graphical flowchart summarizing the study workflow.

The study workflow consisted of the following five protocols: (1) medical image processing and modeling of patient-specific orbital implants, (2) construction of PEEK orbital mesh implant design variants, (3) construction of computational models, (4) AM processes for PEEK orbital mesh implants, and (5) multi-criteria decision-making (MCDM)—configuration assessment. Figure 1 illustrates a graphical flowchart summarizing the study workflow.

### 2.1. Medical Image Processing and Modeling of Patient-Specific Orbital Implants

**Figure 2:**
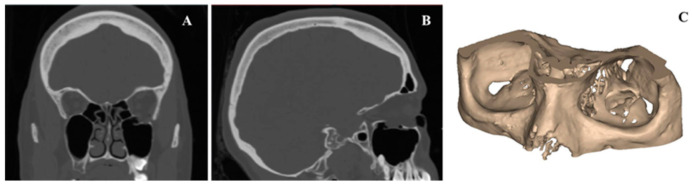
Medical image processing for the generation of a three-dimensional (3D) volumetric reconstruction of an exemplary case with left-sided orbital floor fracture. (**A**) Coronal view. (**B**) Sagittal view. (**C**) 3D volumetric reconstruction of the orbital region of interest.

An anonymized Digital Imaging and Communications in Medicine (DICOM) dataset of a unilateral orbital floor trauma was selected from the hospital’s database for this workflow. A standard high-resolution computed tomography (CT) (Siemens SOMATOM, Siemens Healthcare GmbH, Erlangen, Germany) for trauma protocol with the following parameters was used (matrix of 512×512 pixels, reconstruction slice thickness 0.75 mm, seed per rotation of 1 mm, gantry tilt 0°, bone window setting). The CT dataset was imported into a medical image processing software (Mimics Innovation Suite v. 22.0, Materialise, Leuven, Belgium). A 3D volumetric reconstruction of the skull model was generated using greyscale threshold-based segmentation, and the orbital region of interest (ROI) was selected (Figure 2).

**Figure 3:**
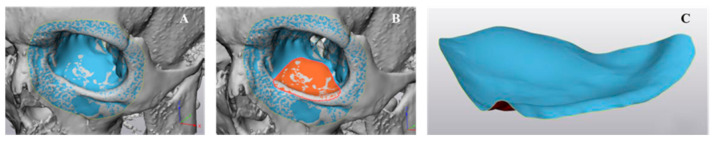
Virtual surgical planning (VSP) for the reconstruction of the orbital implant. (**A**) Mirroring the unaffected (blue) orbit to the contralateral side, i.e., the fractured side (grey), results in a fractured orbital floor’s surface reconstruction. (**B**) Digital reconstruction of the orbital implant with smooth contour continuity. (**C**) Surface model of the customized orbital implant.

The 3D virtual orbital model was then imported into certified CAD software for design modeling (3-matic Medical v. 14.0, Materialise, Leuven, Belgium). The unaffected (non-fractured) orbit was mirrored onto the contralateral side, i.e., the defect/fractured side. Due to fragile bony structures in orbit, all the air spaces were digitally reconstructed using fill hole freeform functionality and a spline-based algorithm (Figure 3A). This resulted in a virtual model of the orbit with smooth contour continuity. The digital reconstruction of the orbital floor was subsequently used as a reference to design the customized orbital implant (Figure 3B). A curve was delineated manually with an extend slightly larger than the orbital bone defect to have stable implant support. This reconstruction resulted in a covering of the defect with zero thickness, hereby referred to as a surface model of the orbital PSI (Figure 3C). This CAD file was saved and exported in the standard tessellation language (STL) file format.

### 2.2. Construction of PEEK Orbital Mesh Implant Design Variants

**Figure 4:**
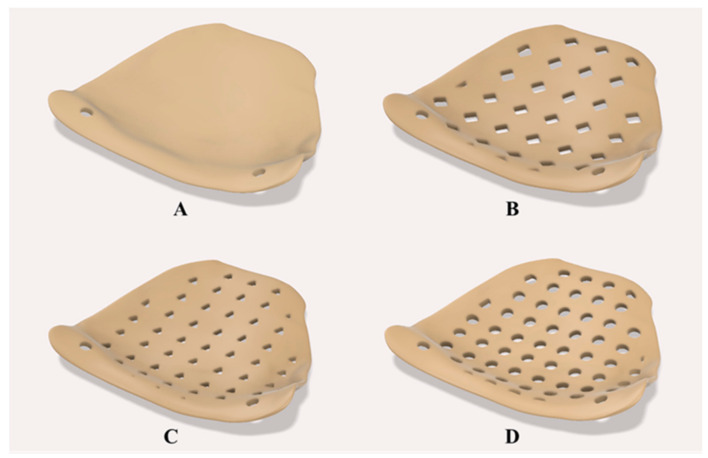
Construction of PEEK orbital mesh implant design variants. (**A**) Solid with no design pattern (reference). (**B**) Rectilinear pattern. (**C**) Triangular pattern. (**D**) Circular pattern.

This step’s main objective was to model thin orbital mesh implants in variable thicknesses and design variants. The surface model of the orbital PSI was imported in another CAD software (Autodesk Inventor v. 2020 for Windows, Autodesk Inc., San Rafael, CA, USA) for the subsequent modeling process. Using “sketch-driven pattern” functionality, three variable design patterns were selected. A solid (no design pattern) model was chosen as a reference, and three different porous constructs, herein referred to as “rectilinear”, “triangular”, and “circular”, were modeled onto the orbital implant (Figure 4). Subsequently, using “mesh enabler” and “convert to freeform” functionalities, the surface model was extruded with appropriate thicknesses. In total, five thickness configurations were chosen for the orbital PSI, and implants with a thickness value of 0.5 mm, 0.6 mm, 0.7 mm, 0.8 mm, and 0.9 mm were modeled. This resulted in a total of 15 orbital PSIs with desired model configuration profiles. For the sake of brevity, these configurations were systematically labeled using a two-character code. The first character in the code (an alphabet) denotes the specific design pattern, and the second character (a number) denotes the thickness of the implant. For instance, a label R07 denotes an implant with a rectangular design pattern and a thickness of 0.7 mm.

### 2.3. Construction of Computational Models

To investigate the structural properties of the configured profiles, finite element (FE) models of the orbital mesh implants were created. The created geometric models of the individual profiles were simplified as shell models and constructed in Inventor Nastran software (Autodesk Inventor Nastran v. 2020 for Windows, Autodesk Inc., San Rafael, CA, USA). The simulation setup was designed to mimic a worst-case scenario in a pure orbital blowout fracture, confined within the internal orbital wall with no supporting bone structure underneath and no fracture involvement of the orbital rims. To design the simulation with clinically relevant forces, a vertical force of 0.3 N was considered, corresponding to the weight of an average eye of 30 g. The loading force was distributed uniformly in a 4 mm diameter circular zone, located in the middle of the implant 10 mm away from the infraorbital rim extent [47,48]. Two screw fixation points with a diameter of 1.5 mm were considered at the infraorbital rim region. All degrees of freedom (DOF) of the nodes around the screw head were constrained. The setup represented a classical cantilever bending test, and therefore, the rotational DOF around the implant was considered free, while the translational DOF was constrained along all axes.

The meshing method is an essential aspect of the FE analysis for design validation since it establishes the accuracy of the investigative results. All models were discretized using a global element size of 0.2 mm. The elements were defined to be parabolic with square elements, i.e., each shell element has eight nodes. To avoid singularities, the configuration models were re-meshed to accomplish regular triangulation. In the area around the screw fixation points, the number of elements was refined and increased by 50 using an additional mesh control. For the rectangular and triangular pattern profile configurations, the mesh size was additionally specified with an element size of 0.01 mm. In contrast, for the circular pattern, a refinement of the mesh size of 0.05 mm was adequate to avoid singularities. These element sizes were chosen based on the preliminary tests and convergence calculations. A sensitivity test was also run to find the best mesh size, which was found when the results showed that the mesh could not be changed by more than 1% across simulations using different mesh densities. The number of elements and nodes used for various design pattern profiles are illustrated in Table 1.

The material properties were defined to be homogeneous, isotropic, and linearly elastic. According to the experimental data in a previous study for characterized properties of PEEK [49], the density of 1.30 g/cm^3^ with the yield strength of 107.1 MPa was taken, the Young’s modulus was set to 4100 MPa, and the Poisson’s ratio of 0.38 was used. The FE analyses were performed, and deformation (in mm) was evaluated along the X, Y, and Z coordinates. Additionally, the von Mises stress was evaluated for each implant profile configuration.

### 2.4. Additive Manufacturing Processes for PEEK Orbital Mesh Implants

The thin orbital mesh implants were fabricated using a material extrusion-based FFF desktop 3D printer, designed specifically for PEEK medical additive manufacturing. The PEEK 3D printer (Apium M220, Apium Additive Technologies GmbH, Karlsruhe, Germany) is designed to generate PSIs in a hospital setting following the International Organization for Standardization (ISO) 10,993 series of requirements for the biological assessment of medical series [50]. The printer includes a temperature control system that offers an enclosed heated environment around the part during the layer-by-layer manufacturing process. The orbital mesh implants were fabricated using a medical-grade 1.75 mm PEEK filament extruded from Vestakeep^®^ i4 G resin (Evonik Vestakeep^®^i4 G resin, Evonik Industries AG, Essen, Germany). This is a high viscosity, natural-colored, and implant-grade material widely used for long-term implantation and fulfills the requirement of the American Society for Testing and Materials (ASTM) F2026-17 guideline for PEEK polymers surgical implant applications [51].

For the fabrication process, the STL files of the designed implant profile configurations were imported into a slicing software (Simplify 3D version 4.0, Cincinnati, OH, USA) compatible with the 3D printer. Due to the complex geometry of the implants, the FFF printing process adds support structures underneath the overhangs and unsupported features. The initial attempts to print the PEEK orbital mesh implants with support structures resulted in poor printability and defective parts. Therefore, a different approach was adopted to fabricate the implants in various design configurations.

**Figure 5:**
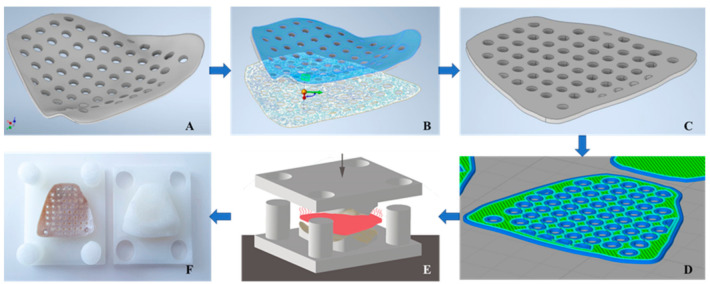
Schematic representation of the workflow implementing a reversed-origami approach for conversion of three-dimensional (3D) computer-aided design implant shape into a 2D structure and 3D printing processes for conversion into a patient-specific orbital mesh implant. (**A**) 3D CAD implant design. (**B**) Unwrapping of the implant’s 3D surface to 2D. (**C**) Thickness offset for the respective implant profile configuration. (**D**) G-code generation with selected printing parameters. (**E**) Thermoforming process. (**F**) Patient-specific 3D PEEK orbital mesh implant.

We implemented a reversed-origami approach by converting the 3D CAD implant shape into a flattened 2D structure (Figure 5). To transfer the model into 2D space, an angular point on the implant surface was selected, and the “unwrap” functionality was used (Autodesk Inventor v. 2020 for Windows, Autodesk Inc., San Rafael, CA, USA). Subsequently, the resulting surface was extruded by the “thickness/offset” function for the selected thickness profile. The flattened implant profile configurations were then printed without any support structures. After printing, the implants were manually separated from the printed raft, and no further post-processing procedures were conducted.

A mold and a press box were created to convert these flattened printed implant configurations back into 3D orbital mesh forms. A lower mold representing the orbital floor plane as its surface and an upper mold with the negative of the orbital floor plane as its counterpart were designed. Finally, a rectangular press box with circular fitting connections was created to assemble the two mold pieces in a rotation- and displacement-free manner. The two-component mold and press-box were fabricated in 3D printed nylon material using a FFF desktop 3D printer (Original Prusa i3 MK3S, Prague, Czech Republic).

Each flattened printed implant was then heated with a hot air gun at 250–300 °C for 5 min to allow easier thermoforming. To prevent structural damage to the implant, it was essential to keep the temperature below PEEK’s melting point of 343 °C [52]. The heated implant was then inserted into the 2-component mold, and the press-box was compressed in a hydropulser at 0.5 MPa. Once cooled down to room temperature, the thin PEEK orbital mesh implant, now formed into a patient-specific 3D designed shape, was removed from the press box. An oral and maxillofacial surgeon independently evaluated the overall visual and tactile fit of the thermoformed PEEK mesh implants in terms of clinical appropriateness for possible orbital floor fracture repair. This evaluation was further quantified into four distinct grades: (1) poor, (2) satisfactory, (3) good, (4) excellent.

### 2.5. Multi-Criteria Decision-Making (MCDM)—Configuration Assessment

To achieve a conclusive result, a performance score for each configuration was devised. Each implant profile configuration was analyzed in terms of stress intensity, deformation, and morphological fit. A weighted assessment factor (AF) was developed to combine the above-mentioned constituent criteria as a metric. This factor was developed using a multi-criteria decision-making (MCDM) method called as Weight Sum Method (WSM) [46].

The stress intensity, deformation patterns, and the morphological fit of the implant were taken as n criteria (C) (Cj for j = 1, 2, 3, …n), while the alternatives (A) were formed by m possibilities (A_i_ for i = 1, 2, …m) according to the pattern profile and thickness value of the implants. The following equation calculated the WSM-score of the i-th alternative:

(1) $${\mathrm{AF}}_{i}=\overset{N}{\underset{j=1}{\sum }}w_{j}\cdot y_{\mathrm{ij}}$$

where AF_i_ is the assessment factor of the i-th alternative, w_j_ is the weighting factor of the j-th criterion, y_ij_ is the value of the i-th alternative for the j-th criterion. As these criteria have different analysis units, the result values were normalized by a linear max-min normalization method [53], as illustrated in the following formula:

(2) $$\frac{r_{j}^{\max}-r_{\mathrm{ij}}}{r_{j}^{\max}-r_{j}^{m_{i}n}}$$

where r_ij_ describes the score of the i-th alternative for the j-th criterion. r_j_^max^ describes the maximum value of a criterion, and r_j_^min^, the corresponding minimum value. Considering a linear relationship between the normalized values and the actual values, the worst value of Cj is assigned a value of 0, and the best value of Cj is assigned a value of 1. After normalization, a weighting factor (w) was assigned to each criterion, which was defined as:

(3) $$w_{j}=\frac{1}{n}$$

The value of each criterion is substantial, and the weighting variables were carefully determined with their total equal to 1. All criteria were deemed to be equally essential, and therefore, in this study, the weighting factors of 0.33 were considered. Lastly, the assessment factor for each implant profile configuration was also normalized by the mean value of all AFs and their standard deviation (SD) for the final comparison using the following equation:

(4) $${\mathrm{AF}}_{i}^{*}=\frac{{\mathrm{AF}}_{i}-\mu }{\sigma }$$

where μ is the AF mean value within the implant profile configuration, and σ is the SD of the assessment factors. The implant profile with the best configuration was represented with a positive AF_i_* and the worst configurations were represented with a negative AF_i_* value.

## 3. Results

Various profile configurations for thin PEEK orbital mesh implants were modeled, simulated, and 3D printed. The results were analyzed for the following quantitative and qualitative criteria, i.e., stress intensity, deformation patterns, and morphological fit of the implants. Utilizing the MCDM technique, the normalized AF_i_* for each implant profile configuration was then calculated to represent the performance score. The AF_i_* value depicted the implant profile configurations, i.e., a higher value of AF_i_* indicated a higher performance score. The individual values of the assessed criteria are reported in the following sections.

### 3.1. Stress Intensity Patterns in the Thin PEEK Orbital Mesh Implants

**Figure 6:**
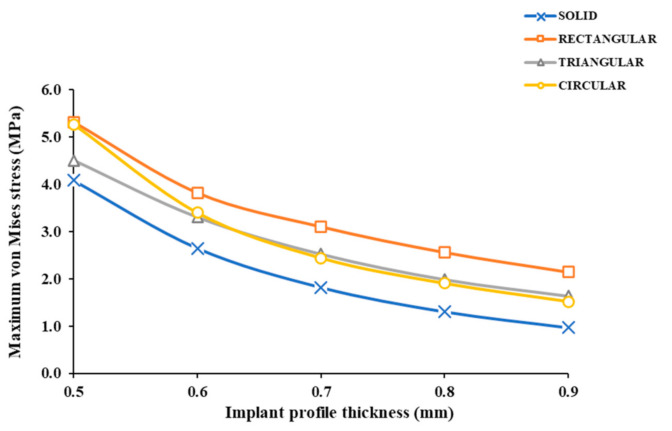
Maximum von Mises stress intensity (MPa) values within each implant profile configuration.

The maximum von Mises stress values in the implants ranged from 1.519 to 5.31 MPa. The typical von Mises stress distribution for the different implant profile configurations is illustrated in Table 2. The thinnest implant profiles resulted in the highest stress values. The highest and the lowest values of maximum von Mises stress were observed in R05 and C09, respectively (Figure 6). The stress intensity plots showed varying levels depending on the design pattern. The stress distribution in the rectangular patterned implants was most pronounced, while in triangular and circular patterned implants, similar distribution was noticed in all, except 0.5 mm profile implants.

### 3.2. Deformation Patterns in the Thin PEEK Orbital Mesh Implants

**Figure 7:**
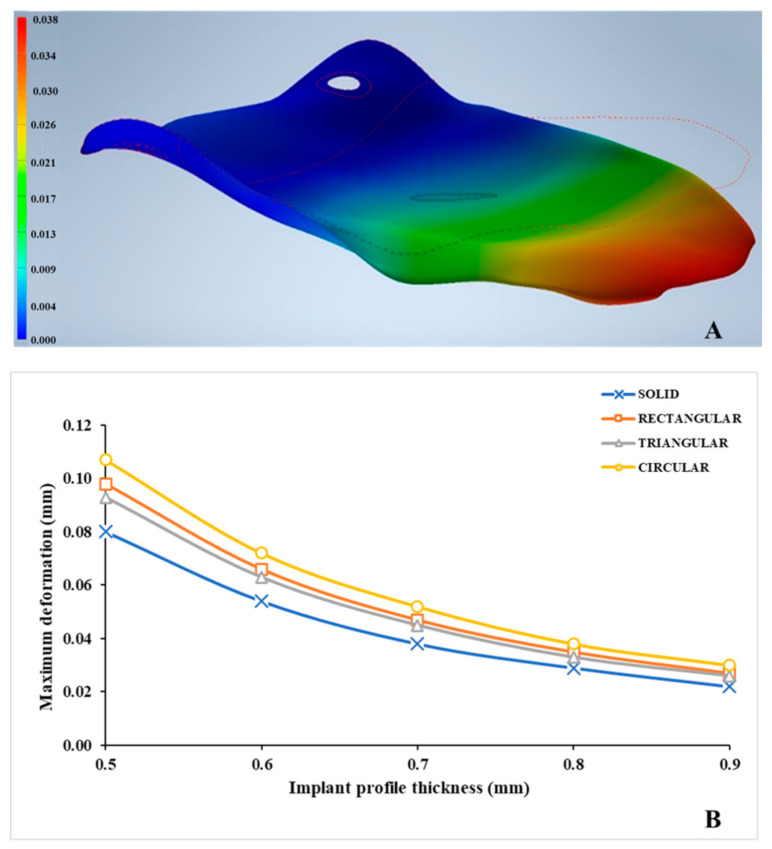
Deformation patterns in the PEEK customized orbital implants. (**A**) Deformation (mm) in a solid (reference) PEEK orbital implant. (**B**) Maximum deformation (mm) within each implant profile configuration.

The deformation values in various implant profile configurations ranged from 0.026 to 0.107 mm, as illustrated in Table 2. Figure 7A illustrates a magnified view displaying the evident orbital implant deformation in a solid implant (reference) profile. The highest and the lowest values for deformation were observed in C05 and T09 configurations, respectively (Figure 7B). It was noticed that with increasing implant thickness, the deformation became less pronounced. All the mesh implants deformed in an anti-clockwise manner with a downward displacement (Z-direction). The deformation was most pronounced at the posterior extent of the implant.

### 3.3. Morphological Assessment of the Thin PEEK Orbital Mesh Implants

**Figure 8:**
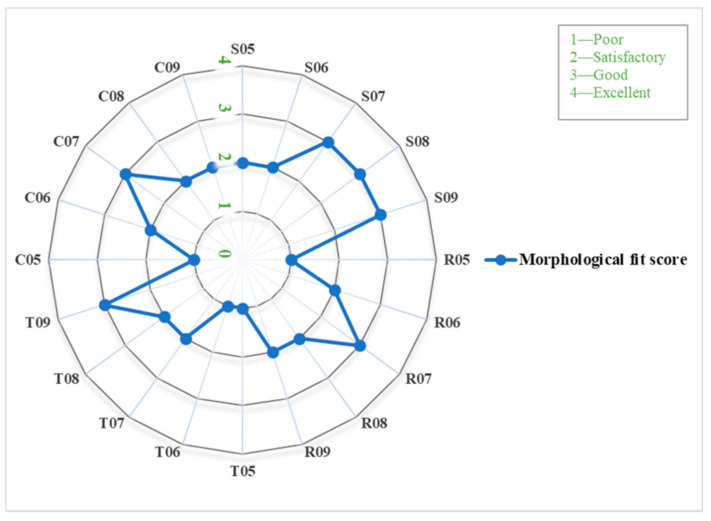
Polar plot representing the morphological fit score (1 to 4) within each implant profile configuration.

The morphological assessment for the overall fit of various implant profile configurations is illustrated in Figure 8. It was noticed that in all the design patterns, implants with a thickness profile of ≥0.7 mm had a higher grade. R07, T09, and C07 profile configurations displayed the highest score with a “good” (3; 20%) grade. The morphological fit of most of the implant profile configurations was rated as “satisfactory” (8; 53.3%) or poor (4; 26.6%).

### 3.4. Configuration Assessment Using Multi-Criteria Decision Making (MCDM) Approach

**Figure 9:**
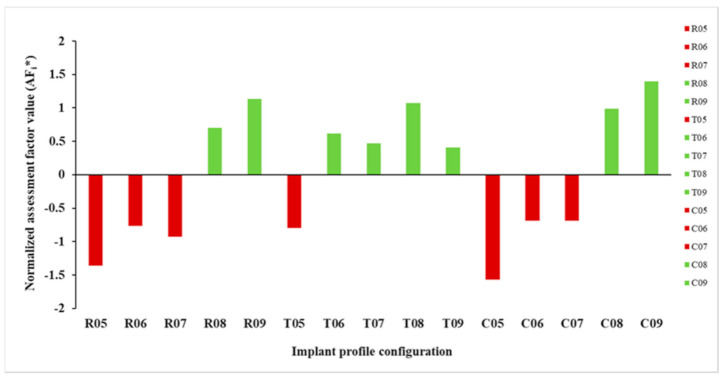
Normalized assessment factors (AF_i_*) within each implant profile configuration.

**Figure 10:**
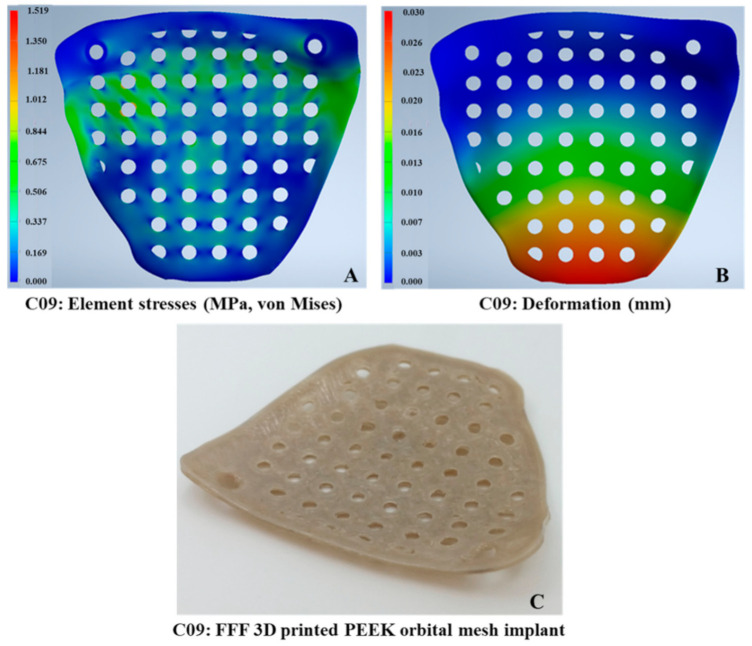
Orbital implant profile configuration with the best performance score. (**A**) C09 stress intensity (MPa) plot. (**B**) C09 deformation (mm) plot. (**C**) C09 material extrusion-based 3D printed PEEK orbital mesh implant.

The computed AF_i_* value for each implant profile configuration is illustrated in Figure 9. It was noticed that in all the design patterns, implants with a thickness profile of ≥0.8 mm had a better performance score with a positive AF_i_* value. All but a 0.5 mm thickness profile revealed a positive AF_i_* value in the triangular patterned implants. The best and worst implant profile configurations, based on AF_i_* value, were C09 and C05, respectively. Figure 10 illustrates the stress intensity and deformation plot for the best implant profile configuration.

## 4. Discussion

The advanced capabilities of 3D CAD modeling and printing technology are changing a wide range of medical specialties, with craniomaxillofacial surgery being one of the most significant benefactors. Custom-made or PSIs are now accessible for various clinical scenarios, allowing for better functional and esthetic outcomes with less surgical time and no donor site morbidity [54]. The use of PEEK PSIs in cranial reconstructions is well documented in the literature, with a trend toward lower implant failure rates with PEEK versus titanium mesh [55]. Furthermore, PEEK PSIs have also been used in craniofacial defects reconstruction [56], in midface reconstruction as an alternative to composite free tissue transfer or maxillary obturators [57,58]. In addition, PEEK onlay implants have been utilized in zygoma contour augmentation [31,32] and mandibular angle reconstructive surgeries [59]. While the use of CAD/CAM milled PEEK orbital implants have been documented in the literature [60,61], the production of porous, mesh-like orbital implants by FFF is relatively new. With improvement in AM systems, the potential for customized FFF 3D printed PEEK implants has surfaced, boosting interest in POC manufacturing [25,26,30].

The development of PSIs at the POC requires the construction of a complete in-house digital workflow. Here, we implemented clinical experience and engineering principles to generate a technical roadmap from preoperative CT datasets, to VSP, to computational models of various design variants, to the fabrication of PEEK PSIs using FFF 3D printing technology. More specifically, a clinical case with patient-specific PEEK orbital reconstruction was evaluated under 0.3 N static load. We then assessed the performance of each implant profile configuration through the WSM-based assessment criteria.

To evaluate the performance of each implant profile configuration, the mechanical response regarding stress and deformation patterns were observed. The maximum von Mises stresses observed in the configurations were in the range of 1.519 to 5.313 MPa. Different stress intensity plots were noticed with increasing implant thickness and changing patterns; however, these differences were not substantial. Although assessing the limit states (e.g., yielding, fatigue) was outside the scope of this study, it is worth noting that stress peak values in all the implant profile configurations were below the assumed material’s yield (failure) stress value (107 MPa). The maximum stress values achieved in our results predict the high durability of the implants, and none of the implant profile configurations exceeded this permissible limit.

In all implant profile configurations, the maximum deformation values were largely under one-tenth of a millimeter (mm). Only one implant profile configuration showed large deformation of 0.107 mm (C05). The individual results revealed that the implant thickness is the most significant factor affecting the stress and deformation patterns in all evaluated configurations. On the contrary, the design patterns had more effect from the fabrication point of view affecting the morphological characteristics. Signs of inaccurate pattern shape were observed in a triangular patterned design. It can be ascertained that the manufacturing process significantly influences the clinical applicability of an implant. The thinner implants, i.e., <0.7 mm, had less thermoforming time and all the implants retained their shape after thermoforming. The assessment factor helped differentiate between configurations and resulted in a composite assessment based on performance score. Furthermore, it was noticed that regardless of the implant thicknesses, the thermoformed PEEK mesh implants maintain the patient-specific shape, and recontouring can only be achieved when re-heated up to 300 °C. As PEEK is a high-temperature thermoplastic biomaterial with good mechanical strength, rigidity, stiffness, and dimensional stability properties [19,27,29,33], the insertion process during surgery must prevent deformation of the mesh contour. During the insertion process, the PEEK mesh implants may require rotation to be adequately positioned for a stable recontouring of the orbital walls. Therefore, adequate retraction of the intra-orbital soft tissues, with no orbital fat or muscles entrapment, should be achieved.

Even though FFF appears to be a straightforward procedure, achieving high efficiency and high-quality manufacturing outputs in PEEK printing presents considerable hurdles [25,43]. Studies have shown that the amount of crystallinity of PEEK material influences its mechanical characteristics. Increasing the crystallinity of a PEEK component can enhance its elastic modulus and yield strength [62]. It was noticed that all the implant profile configurations displayed optimal crystallinity with no visible signs of amorphous (dark-colored) regions. Due to the inherent printing mechanism of FFF, another aspect that needs to be considered is the support structures. The fabrication of thin, complex-shaped PEEK implants with support structures contributes to extensive post-processing procedures and results in a rougher implant surface. This aspect can limit the clinical applicability of an implant. Therefore, an alternative approach was considered in this study to manufacture implants with minimal post-processing and without any support structures. The results in our study validate that a straightforward thermoforming procedure can be applied for PEEK customized mesh implants.

**Figure 11:**
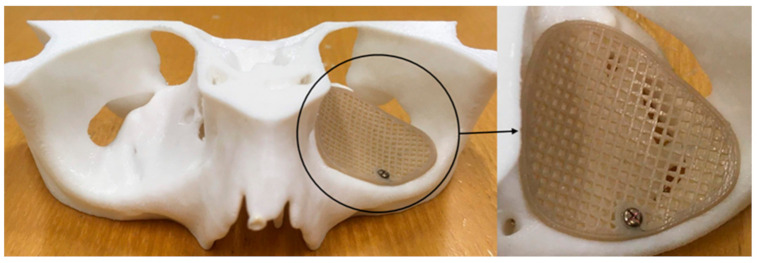
3D printed biomodel with material extrusion-based 3D printed PEEK orbital mesh implant (0.7 mm) fabricated using the rectangular infill pattern.

Although AM offers design freedom meaning that very complex designs are feasible to manufacture, the printing mechanism of the selected AM technology is often a decisive factor. A specific design for one AM technology might not be suitable for another printing technology. Therefore, principles of design for AM (DfAM) must always be taken into consideration. Design for AM (DfAM) methods seek to fully exploit the inherent functionalities of a printing technology resulting in improved performance of biomaterials. Several unseen combinations with beneficial properties can be generated, resulting in sophisticated geometrical designs [63,64,65,66]. We implemented an “infill-based” approach to fabricate the PEEK customized orbital mesh implant to comprehend this aspect further. A g-code based tool path was created using the orbital implant STL file with no pattern. The in-built infill-pattern functionality in the slicing software was used. Figure 11 illustrates a 0.7 mm orbital PEEK mesh implant fabricated using the rectangular infill pattern. This paradigm represents the applicability of in-built printing functionalities and motivates further research to investigate the intrinsic FFF 3D printing characteristics.

The study limitations include simplification of the FE computational model. The use of nonlinear analyses and volume elements was initially computed in this study. However, the results did not reveal significant differences compared to the linear static analysis. Therefore, considering short computational time, linear static analyses were used, which is helpful for a faster comparison of various implant profile configurations. Another significant area of concern is the anchoring capabilities of the fixation screws and the intrinsic heterogeneity in bone quality [67,68]. Substantial high stresses at the screw-bone interface can jeopardize the overall implant stability. Such an estimation of the implant profile configurations would require detailed analysis, particularly at the component interfaces. Therefore, the FE analysis in our study represents a nominal value. However, considering the study objective, the simulation setup in this work, on the other hand, demonstrates an effective technique in the relative evaluation of various design profiles while avoiding complicated model setup and computational cost. Moreover, the approach can be further improved by integrating anisotropic material characteristics of FFF 3D printed parts. Furthermore, we confined the research variables to three criteria; however, the weighting factors can be further tailored to the unique requirements of the analysis. Lastly, we evaluated the performance in one clinical scenario case; further studies are needed to assess the performance in defects with increasing complexity.

## 5. Conclusions

With CAD and 3D printing, multiple treatment options can be devised. An implant for orbital floor reconstruction should minimize the extreme stresses and deformation under physiological conditions and have optimal printing characteristics from a clinical perspective. The study provides insights into the concept of POC FFF 3D printing of PEEK orbital mesh customized implants. Using MCDM, FE-based computational analysis, and FFF 3D printing can be evaluated in multiple treatment options. This approach demonstrates that a range of combinations can be assessed to reach the most effective clinical solution.

## Figures and Tables

**Table 1:** The number of elements and nodes in various design pattern profiles.

	Solid	Rectangular	Triangular	Circular
Number of elements	18,883	26,806	31,915	26,474
Number of nodes	57,124	82,296	97,995	83,036

**Table 2:** Stress and deformation pattern in various implant profile configurations.

Implant Profile Configuration	Max. Von Mises (MPa)	Max. Deformation (mm)
R05	5.313	0.098
R06	3.821	0.066
R07	3.104	0.047
R08	2.563	0.035
R09	2.147	0.027
T05	4.502	0.093
T06	3.304	0.063
T07	2.522	0.045
T08	1.986	0.033
T09	1.636	0.026
C05	5.267	0.107
C06	3.397	0.072
C07	2.439	0.052
C08	1.904	0.038
C09	1.519	0.030

## Data Availability

The original contributions presented in the study are included in the article, further inquiries can be directed to the corresponding author.

## Abbreviation

2D	Two-Dimensional
3D	Three-Dimensional
AF	Assessment Factor
AM	Additive Manufacturing
ASTM	American Society for Testing and Materials
CAD	Computer-Aided Design
CAM	Computer-Aided Manufacturing
CAS	Computer-Assisted Surgery
CT	Computed Tomography
DfAM	Design for Additive Manufacturing
DICOM	Digital Imaging and Communications in Medicine
DOF	Degree of Freedom
FE	Finite Element
FFF	Fused Filament Fabrication
ISO	International Organization for Standardization
MCDM	Multi-Criteria Decision Making
PEEK	Polyetheretherketone
POC	Point-of-Care
PSIs	Patient Specific Implants
ROI	Region of Interest
SD	Standard Deviation
STL	Standard Tessellation Language
VSP	Virtual Surgical Planning
WSM	Weight Sum Method
